# Supplementary material for: Breastfeeding Practice and Association between Characteristics and Experiences of Mothers Living in Bangkok
Source: Int J Environ Res Public Health. 2021 Jul 26;18(15):7889. doi: 10.3390/ijerph18157889 (PMC8345453; doi:10.3390/ijerph18157889)
Supplement: Supplementary file 1 [file ijerph-18-07889-s001.zip › ijerph-1282286-supplementary.pdf]

Supplementary file

Table S1. The full list of hospitals and health centers with a corresponding number of samples acquired.

Health facilities	Number	Freq.	Percent	Cum.
Hospital	1	195	28.85	28.85
Hospital	2	63	9.32	38.17
Hospital	3	22	3.25	41.42
Hospital	4	19	2.81	44.23
Hospital	5	45	6.66	50.89
Hospital	6	43	6.36	57.25
Hospital	7	51	7.54	64.79
Hospital	8	71	10.50	75.29
Health Center	1	1	0.15	75.44
Health Center	2	7	1.04	76.48
Health Center	3	19	2.81	79.29
Health Center	4	1	0.15	79.44
Health Center	5	9	1.33	80.77
Health Center	6	4	0.59	81.36
Health Center	7	14	2.07	83.43
Health Center	8	39	5.77	89.20
Health Center	9	4	0.59	89.79
Health Center	10	2	0.30	90.09
Health Center	11	20	2.96	93.05
Health Center	12	1	0.15	93.20
Health Center	13	45	6.66	99.85
Health Center	14	1	0.15	100
